# Induction of protective proteins is fundamental to hyperbaric oxygen preconditioning

**DOI:** 10.3389/fcell.2026.1831774

**Published:** 2026-05-18

**Authors:** Jiahe Zhou, Quan Zhou, Qi Zhu, Danfeng Fan, Xiaoxiao Ni, Long Qing, Guoyang Huang, Weigang Xu

**Affiliations:** 1 Department of Diving and Hyperbaric Medicine, Naval Medical Center, Naval Medical University, Shanghai, China; 2 Department of Hyperbaric Oxygen, the Sixth Medical Center of PLA General Hospital, Beijing, China; 3 Department of Hyperbaric Oxygen and Rehabilitation, General Hospital of Southern Theater Command of PLA, Guangzhou, China

**Keywords:** HIF-1α, HSF1, hyperbaric oxygen preconditioning, Nrf2, protective proteins, reactive oxygen species

## Abstract

Hyperbaric oxygen (HBO) therapy involves the administration of 100% oxygen at elevated atmospheric pressure and has long been utilized for both therapeutic and prophylactic purposes. While early applications of HBO pretreatment primarily emphasized denitrogenation and denucleation to prevent decompression sickness in diving and aerospace settings, accumulating preclinical studies and clinical evidence now indicate that HBO pretreatment’s broader protective effects are mediated by preconditioning. Central to this process is the generation of reactive oxygen species (ROS), which serve as critical signaling molecules that trigger adaptive cellular responses upon exposure to HBO. ROS induced by HBO activates key transcriptional regulators, including nuclear factor erythroid 2-related factor 2, heat shock factor 1, and hypoxia-inducible factor-1α, leading to the upregulation of protective proteins such as heme oxygenase-1, heat shock proteins, and vascular endothelial growth factor. These molecular responses enhance tissue resilience, promote cellular survival, and improve tolerance to ischemic, inflammatory, and oxidative injuries observed across diverse clinical conditions. This review focuses on HBO-induced protective proteins, synthesizing current knowledge of their molecular mechanisms, expression dynamics, and clinical relevance, as well as the role of exposure protocols in regulating their expression. The findings provide a framework for designing and interpreting future clinical applications of HBO preconditioning.

## 1 Introduction

The physiological and therapeutic benefits of hyperbaric oxygen (HBO) therapy have been demonstrated not only in hyperbaric or hypobaric environmental conditions but also in a range of clinical applications (Jones et al., 2024). The Undersea and Hyperbaric Medical Society currently recognizes HBO as an approved intervention for approximately 15 disorders, including arterial gas embolism, decompression sickness (DCS), carbon monoxide poisoning, and stroke (Ortega et al., 2021; Weaver, 2014). The 10th European Consensus Conference on Hyperbaric Medicine further expanded the list of indications to 31 distinct conditions (Mathieu et al., 2017). In China, the clinical application of HBO has been extended to include acute coronary syndrome, chronic peripheral vascular insufficiency, high-altitude maladaptation, and numerous other disorders (Hospital, 2019). Beyond its therapeutic roles, HBO has also demonstrated preventive efficacy across a variety of settings, including the preoperative period, diving preconditioning, and high-altitude acclimatization (Alex et al., 2005; Camporesi and Bosco, 2014; The Sixth Medical Center of PLA General Hospital and Western Theater General Hospital, 2025). These findings demonstrate that HBO has expanded from a therapeutic intervention to a preventive strategy against both clinical operations and environmental exposures. Despite these beneficial effects, it is important to recognize that oxygen at high partial pressures, typically above 3 ATA, may exert toxic effects on biological tissues. High-pressure oxygen exposure can induce central nervous system oxygen toxicity manifesting as seizures, as well as pulmonary oxygen toxicity characterized by oxidative damage and impaired respiratory function. These adverse effects are closely related to excessive oxidative stress, emphasizing the importance of appropriate pressure selection and exposure duration in HBO application (Louise Thomson and Paton, 2014; Camporesi, 2014).

The earliest research on HBO for disease prevention dates back to denitrogenation before flight in pilots and extravehicular activities in astronauts, as well as denucleation before diving. Denitrogenation is a process of eliminating nitrogen from the body by achieving adequate preoxygenation, thereby preventing bubble formation during or following a decrease in ambient pressure (Carmichael et al., 1989). A study showed that 1 h of preoxygenation followed by 4 h of exposure to 0.3 ATA reduced the risk of DCS from 77%–47% (Webb and Pilmanis, 1998). NASA determined that 3 h of normobaric oxygen denitrogenation was essential for preventing DCS during extravehicular activities (EVA) (Tokumaru, 1997). The denitrogenation strategy is also applicable to civilian and military aviation personnel who frequently encounter low-pressure exposure, submarine escape, and saturation diving (Eiken et al., 2023; Latson et al., 2000). The formation of bubbles from supersaturated dissolved gases requires the presence of gas nuclei (Blatteau et al., 2006; Evans and Walder, 1969). Oxygen diffuses into these micronuclei and is subsequently absorbed by the surrounding tissues, a process described as “denucleation” (Blatteau et al., 2006; Arieli et al., 2002). Reducing the number and the size of gas nuclei can effectively inhibit bubble generation, thereby decreasing the risk of DCS (Arieli, 2017; Arieli and Marmur, 2011). Arieli and his colleagues demonstrated that a 20-min air interval after HBO was more effective than a 2-h interval before high-pressure exposure in reducing the incidence of DCS in rats (Arieli et al., 2002; Ertracht et al., 2005). As the air interval extended beyond 6 h, the protective effect of HBO declined, likely due to the regeneration of gas nuclei (Arieli et al., 2011; Katsenelson et al., 2007).

A growing body of evidence suggests that HBO pretreatment also exerts protective effects in other diseases. In 2005, Alex et al. observed that pretreatment with HBO before on-pump coronary artery bypass grafting (CABG) surgery reduced neuropsychometric dysfunction and modulated the inflammatory response after cardiopulmonary bypass (Alex et al., 2005). Yogaratnam, et al. reported that HBO before on-pump CABG surgery improved left ventricular stroke work post-CABG surgery while reducing intraoperative blood loss, ICU length of stay, and postoperative complications (Yogaratnam et al., 2010). Li, et al. found that HBO pretreatment decreased the release of cerebral and myocardial biochemical markers, including S100B protein, NSE, and cTnI during the peri- and post-CABG surgery period (Li et al., 2011a). These findings indicate that the benefits of HBO extend beyond conventional denitrogenation and gas nucleation suppression. Accordingly, research now focuses on HBO preconditioning as a key mechanism that activates intrinsic protective responses.

## 2 HBO preconditioning

Preconditioning is the application of a mild stimulus to activate endogenous protective mechanisms, thereby ameliorating the morphologic and functional sequelae of a subsequent sublethal insult, such as ischemic, hypoxic, or thermal stress (Camporesi and Bosco, 2014; Liu et al., 2022; Torma et al., 2019). Preoxygenation was first described in a rat model of paraquat-induced neutrophilic pneumonia in 1986 (Martin and Howard, 1986). A decade later, Wada *et al.* demonstrated that HBO preconditioning upregulated hippocampal heat shock protein (HSP) 72 and conferred ischemic tolerance in gerbils (Wad et al., 1996). Since then, extensive research in HBO preconditioning has demonstrated its benefits in promoting ischemic tolerance across various animal models of injury (Li et al., 2007; He et al., 2011; Yan et al., 2013). HBO has now been used in patients before exposure to clinical situations with beneficial effects, but its underlying mechanisms of action remain incompletely understood.

## 3 ROS is the trigger for HBO preconditioning efficacy

Reactive oxygen species (ROS) are generated as natural byproducts of cellular metabolism and encompass superoxide anion, hydroxyl radical, and hydrogen peroxide. These bioactive molecules function as essential mediators in cellular redox signaling pathways, orchestrating various physiological and pathological processes (Hong et al., 2024; Dong et al., 2016). Mitochondria serve as the principal sites of intracellular oxygen consumption, where molecular oxygen is utilized in tightly regulated oxidative phosphorylation to produce adenosine triphosphate (Cash et al., 2007). During electron transport, premature electron leakage can generate ROS. Under hyperoxic conditions, the elevated oxygen partial pressure enhances mitochondrial oxygen availability, providing abundant substrates for ROS formation. Consequently, hyperoxia accelerates aerobic respiration, electron flux, and electron leakage, thereby amplifying ROS production (Gottfried et al., 2021).

To further clarify the main source of intracellular ROS under HBO, we utilized mitochondrial-targeted redox fluorescent probes and specific inhibitors to assess the source of cellular ROS (Zhou et al., 2018). Intracellular ROS levels in endothelial cells increased by approximately 32%–42% during 2.8 ATA HBO exposure, primarily originating from the mitochondrial respiratory chain. In contrast, ROS generated by NADPH oxidase and xanthine oxidase outside the mitochondria were not significantly increased under HBO conditions (Zhou et al., 2018), consistent with previous reports (Schottlender et al., 2021; Angel et al., 1994). The mechanism by which HBO influences mitochondrial function and regulates ROS production has been well reviewed by Schottlender N, et al. (Schottlender et al., 2021).

In 2009, Professor Thom first proposed that oxidative stress is fundamental to hyperbaric oxygen therapy (Thom, 2009). HBO induces moderate ROS production, upregulating both enzymatic and non-enzymatic antioxidant systems, thereby directly limiting oxidative damage while enhancing anti-inflammatory capacity, inhibiting apoptosis, and promoting proliferation and angiogenesis (Thom, 2011). Elevated ROS levels initiate transduction cascades, or pathways, that induce the expression of downstream cytoprotective proteins (Thom, 2009). By the same token, ROS is also a fundamental factor for the prophylactic effect of HBO preconditioning. ROS scavenger N-acetylcysteine (NAC) eliminated the protective effect of HBO on oxidative insult to rat spinal cord neurons (Huang et al., 2016). Both Mito-TEMPO (mitochondrial ROS inhibitor) and NAC suppressed the HBO-induced enhanced resistance in vascular endothelial cells (Xu et al., 2025). Additionally, a study has shown that NAC counteracted the protective effect of HBO preconditioning against hyperglycemia-enhanced hemorrhagic transformation in rats (Soejima et al., 2013). These findings confirm that ROS is an indispensable upstream trigger for HBO-induced protection.

## 4 Protective proteins are the key effectors of HBO preconditioning

**FIGURE 1:**
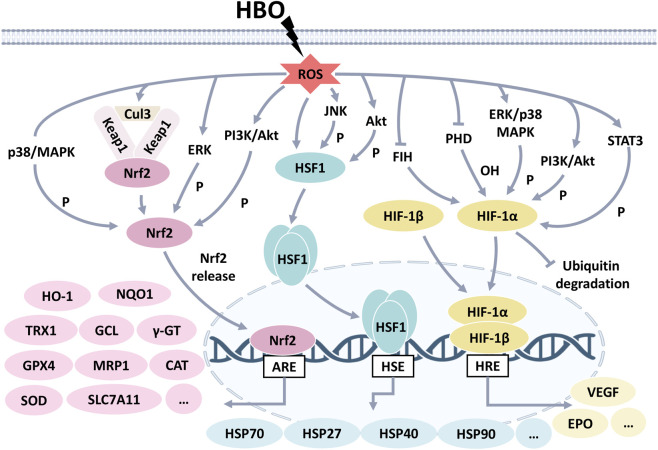
Signaling pathways underlying HBO. ROS works as the trigger of HBO, further activating transcription factors Nrf2 and its downstream proteins (HO-1, NQO1, TRX1, GCL, γ-GT, GPX4, MRP1, CAT, SOD, SLC7A11), HSF1 and its downstream proteins (HSP70/27/40/90), as well as HIF-1 and its downstream proteins (EPO, VEGF), respectively.

HBO increases the expression of various protective proteins. The activation of transcription factors Nrf2, HSF1, and HIF-1 predominantly mediates these proteins. The signaling pathways are illustrated in Figure 1.

### 4.1 Nrf2 activation and downstream protein induction

Nrf2 is a member of the Cap’n’Collar subfamily of leucine zipper transcription factors, involved in antioxidant reactions, NADPH regeneration, heme and iron metabolism (Tonelli et al., 2018). Under physiological conditions, Nrf2 is sequestered in the cytoplasm by the negative regulator, Kelch-like ECH-associated protein 1 (Keap1), which promotes its polyubiquitination and degradation by the Cullin 3 E3 ubiquitin ligase (Ngo and Duennwald, 2022). When cells are exposed to HBO, elevated ROS levels oxidize critical cysteine residues on Keap1 (Bai et al., 2018; Xue et al., 2016; Zhai et al., 2016; Xu et al., 2014; Liu et al., 2025). This oxidative modification triggers a conformational change in Keap1, thereby disrupting its ability to mediate Nrf2 ubiquitination and stabilizing Nrf2, promoting its nuclear accumulation. Within the nucleus, Nrf2 heterodimerizes with small Maf proteins and binds to the antioxidant response element, initiating the transcription of antioxidant enzymes (Xu et al., 2014; Kasai et al., 2020). Moreover, transcriptional activation of Nrf2 can also be stimulated by HBO through PI3K/Akt, ERK, and p38 MAPK signaling cascades (Huang et al., 2016; Huang et al., 2014; Papaiahgari et al., 2006).

#### 4.1.1 Heme Oxygenase-1 (HO-1)

HO-1, also termed heat shock protein 32 due to the heat shock element in its promoter, is an inducible enzyme responsible for the degradation of endogenous protoporphyrin iron heme. The enzymatic activity of HO-1 yields three main bioactive products: carbon monoxide, Fe^2+^, and bilirubin, which exert pro-angiogenic, antioxidant, anti-apoptotic, and anti-inflammatory effects (Silva et al., 2022; Loboda et al., 2016; Syapin, 2008). HO-1 is a direct transcriptional target of Nrf2 (Zhao et al., 2020), The Nrf2/HO-1 axis activated by HBO has emerged as a fundamental defense pathway against oxidative stress in various pathological conditions (Yang et al., 2023; Tripathi et al., 2023; Luo et al., 2021; Zhang et al., 2021), including central nervous system (CNS) ischemia-hypoxic injury (Huang et al., 2016; Zhai et al., 2016), myocardial ischemia-reperfusion (I/R) injury (Liu et al., 2025; Yin et al., 2015), acute lung injury (Bai et al., 2018; Yu et al., 2016) and bronchopulmonary dysplasia (Chu et al., 2020).

#### 4.1.2 NAD(P)H: quinone oxidoreductase-1 (NQO1)

NQO1, another Nrf2-regulated gene, catalyzes the two-electron reduction of both endogenous and xenobiotic quinones using flavin adenine dinucleotide as cofactor, thereby averting redox cycling and oxidative damage (Ross and Siegel, 2017). HBO upregulates NQO1 expression in LPS-induced acute lung injury (Bai et al., 2018). In a traumatic brain injury model, HBO preconditioning reduced neuronal apoptosis and improved neurological outcome by elevating NQO1 (Meng et al., 2016). Moreover, intestinal oxidative stress injury after spinal cord injury was ameliorated by increased NQO1 expression induced by HBO (Liu et al., 2021). Our unpublished data indicated that NQO1 expression in rat pulmonary microvascular endothelial cells was enhanced by HBO, thereby counteracting bubble-induced injury in DCS.

#### 4.1.3 Other Nrf2-Mediated proteins

The protective role of Nrf2 activation by HBO extends beyond HO-1 and NQO1 to multiple downstream proteins. In astrocytes, Nrf2 upregulation enhances the expression of glutamate-cysteine ligase (GCL), γ-glutamyl transferase (γ-GT), and multidrug resistance protein-1 (MRP1), which contribute to spinal cord ischemic tolerance (Xu et al., 2014). In pulmonary microvascular endothelial cells, HBO induced thioredoxin-1 (TRX1) via Nrf2 activation, thereby alleviating bubble-related injury (unpublished data). Moreover, HBO promoted the expression of several key Nrf2-regulated antioxidant enzymes, including superoxide dismutase (SOD), catalase (CAT), glutathione (GSH), glutathione peroxidase 4 (GPX4), and solute carrier family seven member 11 (SLC7A11). These factors not only exert potent antioxidative effects that alleviate intestinal epithelial barrier dysfunction, but also protect HT22 and PC12 cells against oxygen-glucose deprivation/reperfusion injury by inhibiting ferroptosis mediated through the Nrf2/System Xc^−^/GPX4 axis (Liu et al., 2021; Chen et al., 2022).

### 4.2 HSF1 activation and downstream protein induction

HSF1 is a key transcription factor that plays a central role in the cellular response to various forms of stress (Li et al., 2017). HSF1 resides in the cytoplasm in an inactive polymeric form. Upon exposure to heat shock or oxidative stress, HSF1 dissociates from its complex, translocates to the nucleus, binds to the heat shock element, and activates transcription and expression of HSPs, thereby exerting cytoprotective effects (Gomez-Pastor et al., 2018). Evidence indicated that HSF-1-null mutant mice were more susceptible to oxidative damage caused by hyperoxia (Malhotra et al., 2002). We observed that HBO induced the nuclear translocation of HSF1 in pulmonary vascular endothelial cells. Treatment with the ROS scavenger mito-TEMPO or the AKT inhibitor MK2206 reversed this phenomenon (Xu et al., 2025).

#### 4.2.1 Heat shock protein 70 (HSP70)

HSP70, an evolutionarily conserved and robustly inducible member of the HSP family, acts as a molecular chaperone that protects cells from oxidative injury (Gullo and Teoh, 2004). HBO preconditioning increases HSP70, thereby reducing hepatic I/R damage and nitric oxide-mediated chondrocyte death (Wu et al., 2018; Ueng et al., 2013). Our previous study has shown that induction of HSP70 expression in spinal cord and lung tissue by HBO mitigated DCS in rat models (Ni et al., 2013). Moreover, the upregulation of HSP70 induced by HBO in lungs can also prevent high-altitude pulmonary edema (Tsai et al., 2014).

#### 4.2.2 Other HSPs

HSP27 is a small HSP that preserves cytoskeletal integrity, promotes proliferation and migration, and inhibits apoptosis (Janowska et al., 2019). HSP40, a HSP70 co-chaperone, stimulates HSP70 ATPase activity and assists in correct protein folding (Kampinga and Craig, 2010). Our recent study found that HBO also induced HSP27 and HSP40, enhanced the resistance of vascular endothelial cells to bubble injury, and mitigated DCS-related damage in rats (Xu et al., 2025).

### 4.3 HIF-1 activation and downstream protein induction

HIF-1 is a heterodimeric transcription factor composed of HIF-1α and HIF-1β (Semenza, 2014). Under normoxia, prolyl hydroxylase domain (PHD) enzymes hydroxylate HIF-1α, enabling von Hippel-Lindau protein (pVHL)-mediated ubiquitination and proteasomal degradation. Under hypoxia, ROS derived from mitochondrial complex III inhibit PHD activity, leading to HIF-1α stabilization. Stabilized HIF-1α then dimerizes with HIF-1β to form transcriptionally active HIF-1, which translocates to the nucleus and drives genes involved in neovascularization, antioxidation, anti-inflammation, and anti-apoptosis (Semenza, 2014; Nguyen and Durán, 2016; Masson et al., 2012). The level of HIF-1α under hyperoxic conditions is currently under debate, as it may vary with parameters including pressure, duration, and repetition after HBO (You et al., 2023; Cheng et al., 2011; Jadhav et al., 2009; Han et al., 2008; Gu et al., 2008; Peng et al., 2008). This difference is likely related to HBO-evoked ROS accumulation (Hong et al., 2024; Liu et al., 2014; Ren et al., 2008; Fratantonio et al., 2021; Balestra et al., 2006), which could activate signaling pathways such as ERK/p38-MAPK, PI3K/Akt and STAT3, further inducing HIF-1 expression (Du et al., 2011; Pawlus et al., 2014; Yang et al., 2009).

#### 4.3.1 Erythropoietin (EPO)

EPO, a target of HIF-1, is recognized for its role in the proliferation, differentiation, and survival of erythroid progenitors (Bunn, 2013) and provides cytoprotection to multiple organs, including the brain, kidney, heart, lung, and retina (Haine et al., 2021). Notably, EPO also acts as a potent antioxidant, anti-apoptotic, and anti-inflammatory effector (Chateauvieux et al., 2011). A study demonstrated that upregulation of EPO by HBO restored the neurological function and reduced the infarct volume in a rat focal cerebral ischemic model (Gu et al., 2008). Another study found that HBO increased the expression of EPO to prevent the changes in blood-brain barrier permeability and brain edema caused by hypoxia exposure (Peng et al., 2008).

#### 4.3.2 Vascular endothelial growth factor (VEGF)

VEGF, another target of HIF-1α, is known to promote angiogenesis, increase vascular permeability, stimulate mitosis and migration, and inhibit apoptosis (Apte et al., 2019; Masoud and Li, 2015). In a rat model of focal cerebral ischemia, HBO upregulated VEGF expression in cerebral astrocytes (Wang et al., 2016). Moreover, HBO preconditioning enhanced VEGF in animal models of partial hepatectomy, cerebral ischemia, and normal or ischemic myocardium, thereby promoting angiogenesis (Han et al., 2008; Ren et al., 2008; Duan et al., 2015).

Taken together, HSPs represent the earliest proteins identified to be induced by HBO. Subsequent research has established Nrf2 as a central transcription factor that mediates the endogenous protective effects of HBO, upregulating multiple antioxidant proteins and thereby establishing a cellular defense system. Research regarding HIF-1 in response to HBO remains limited and contradictory; thus, further experiments are required to clarify the change and underlying mechanisms under HBO exposure.

## 5 Expression characteristic of HBO-induced proteins

The expression of proteins induced by HBO varies among species, tissues, and cell types, and changes over time. Understanding these characteristics of change is important for understanding the effects of HBO preconditioning in specific pathologies. Based on existing evidence, the expression patterns of protective proteins are summarized in Tables 1–3.

**TABLE 1:** Summary of Nrf2-mediated protective protein induction by HBO.

Transcription factors	Effect proteins	Subjects		HBO protocol			Signaling pathways	References
		Cells/Tissues	Species	Pressure/ATA	Duration/min	Repetition		
Nrf2	HO-1	Spinal neurons	Rat	2.8	60	1	​	Huang et al. (2016), Huang et al. (2014)
		Spinal neurons	Mouse	3.5	120	1	ROS/p38/MAPK	Li et al. (2007)
		Hippocampus astrocytes	Mouse	2.5	60	5	Sirt1	Hu et al. (2018)
		Brain tissues	Rat	2.5	60	5	Sirt1	(Xue et al., 2016; Soejima et al., 2012;)
		Hippocampal tissues	Mouse	2.5	60	5	Sirt1	Hu et al. (2018)
		Myocardial tissues	Mouse, Rat	2.0	60	4	Mst1, PI3K/Akt	Liu et al. (2025), Yin et al. (2015)
		Microvascular endothelial cells	Human	2.4	60	1	​	Godman et al. (2010)
		Lung tissues	Rat	2.8	60	5	​	Bai et al. (2018)
		Lung tissues	Rat	2.0	60	4	​	Feng et al. (2015)
		Lung tissues	Rat	2.0	60	3	​	Yu et al. (2016)
		Kidney tissues	Rat	2.0	60	4	​	Nesovic et al. (2021), Kovacevic et al. (2021)
		Kidney tissues	Rat	2.5	60	4	​	He et al. (2011)
		Kidney tissues	Rat	3.0	120	1	​	Chang et al. (2006)
		Liver tissues	Rat	3.0	120	1	​	
		Liver tissues	Rat	2.5	20	4	​	Liu et al. (2011)
	SOD	Spinal neurons	Mouse	3.5	120	1	ROS/p38/MAPK	Li et al. (2007)
		Spinal cord	Rabbit	2.5	60	5	​	Nie et al. (2006)
		Brain tissues	Rat	2.5	60	5	​	Yan et al. (2013), Xue et al. (2016), Soejima et al. (2012)
		Lung tissues	Rat	2.0	60	3	​	Yu et al. (2016)
		Kidney tissues	Rat	2.5	60	4	​	He et al. (2011)
	NQO1	Pulmonary microvascular endothelial cells	Rat	2.8	60	1	​	Unpublished data
		Lung tissues	Rat	2.8	60	1	​	Unpublished data
		Lung tissues	Rat	2.8	60	5	​	Bai et al. (2018)
	TRX1	Pulmonary microvascular endothelial cells	Rat	2.8	60	1	​	Unpublished data
		Lung tissues	Rat	2.8	60	1	​	Unpublished data
	CAT	Spinal cord	Rabbit	2.5	60	5	​	Nie et al. (2006)
	GCL	Spinal astrocytes	Mouse	2.5	90	1	​	Xu et al. (2014)
	γGT	Spinal astrocytes	Mouse	2.5	90	1	​	
	MRP1	Spinal astrocytes	Mouse	2.5	90	1	​	
	​	PC12 cells	Rat	2.5	60	1	​	Chen et al. (2022)
	​	HT22 cells	Mouse	2.5	60	1	​	Chen et al. (2022)
	​	C10 cells	Mouse	1.0	30/60/180	1	ROS-EGFR-PI3K-Akt/ERK MAPK	Papaiahgari et al. (2006)
	​	A549 cells	Human	1.0	30/60/180	1		Papaiahga et al. (2004)

**TABLE 2:** Summary of HSF1-mediated protective protein induction by HBO.

Transcription factors	Effect proteins	Subjects		HBO protocol			Signaling pathways	References
		Cells/Tissues	Species	Pressure/ATA	Duration/min	Repetition		
HSF1	HSP70	Neuroblastoma cells	Mouse	3.0	60	1–5	​	Shyu et al. (2004)
		Spinal cords	Rat	2.8	60	1	JNK	Ni et al. (2013)
		Hippocampal tissues	Rat	2.0	60	5	​	Lin et al. (2012)
		Lymphocytes	Swine	2.8	60	1	​	Qing et al. (2018)
		Mononuclear cells	Human	2.8	60	1	​	Vince et al. (2011)
		Lymphocytes	Human	2.5	60	1	​	Dennog et al. (1999)
		Myocardial tissues	Human	2.4	30	2	​	Jeysen et al. (2011)
		Lung tissues	Rat	2.0	60	5	​	Tsai et al. (2014)
		Lung tissues	Rat	2.8	60	1	​	Ni et al. (2013)
		Liver tissues	Rat	2.0	60	5	​	Wu et al. (2018)
		Liver tissues	Rat	2.5	45/90/120	1–3	​	Yu et al. (2005)
	HSP90	Spinal neurons	Rat	2.8	60	1	​	Huang et al. (2014)
		Lymphocytes	Swine	2.8	60	1	​	Qing et al. (2018)
	HSP27	Spinal neurons	Rat	2.8	60	1	​	Huang et al. (2014)
		Pulmonary microvascular endothelial cells	Rat	2.8	60	1	ROS/Akt	Xu et al. (2025)
		Myocardial tissues	Rat	2.8	60	1	​	Unpublished data
		Lungs tissues	Rat	2.8	60	1	ROS/Akt	Xu et al. (2025)
	HSP40	Pulmonary microvascular endothelial cells	Rat	2.8	60	1	ROS/Akt	
		Lungs tissues	Rat	2.8	60	1		
	eNOS	Myocardial tissues	Human	2.4	30	2	​	Jeysen et al. (2011)

**TABLE 3:** Summary of HIF-1α-mediated protective protein induction by HBO.

Transcription factors	Effect proteins	Subjects		HBO protocol			Signaling pathways	References
		Cells/Tissues	Species	Pressure/ATA	Duration/min	Repetition		
HIF-1α	VEGF	Myocardial tissues	Rat	2.5	60	4	ROS	Han et al. (2008)
		Liver tissues	Rat	2.5	60	3		Ren et al. (2008)
		Flaps	Rat	2.0	60	6		Liu et al. (2014)
	​	Pulmonary microvascular endothelial cells	Rat	2.8	60	1		Unpublished data
	EPO	Brain tissues	Rat	2.0	60	5		Gu et al. (2008), Peng et al. (2008)
	COX-2	Brain tissues	Rat, Mouse	2.5	60	5		Cheng et al. (2011), Jadhav et al. (2009)
	MMP-2	Brain tissues	Rat	2.5	60	1		Soejima et al. (2013)
	MMP-9	Brain tissues	Rat	2.5	60	1		​
	SDF-1	Flaps	Rat	2.0	60	6		Liu et al. (2014)
	CXCR4	Mononuclear cells	Human	1.0/1.4	60	1		Fratantonio et al. (2021)
		Kidney tissues	Rat	2.0	60	4		Nesovic et al. (2024)
		Flaps	Rat	2.0	60	6		Liu et al. (2014)

### 5.1 Across different species

Based on the available literature, the protective proteins HO-1, SOD, and HSP70 are repeatedly detected in experimental animal models (e.g., mice, rats, rabbits, humans), indicating that the cytoprotective response to HBO is evolutionarily conserved. Specifically, HO-1 is induced by HBO in the CNS and myocardium of rats and mice (Liu et al., 2022; Li et al., 2007; Huang et al., 2016; Soejima et al., 2013; Liu et al., 2025; Huang et al., 2014; Yin et al., 2015; Hu et al., 2018;), and SOD is upregulated in the CNS of mice, rats, and rabbits (Li et al., 2007; Yan et al., 2013; Xue et al., 2016; Soejima et al., 2012; Nie et al., 2006). HSP70 is elevated in peripheral lymphocytes of Bama-pig, a result consistent with observations in human volunteers (Ni et al., 2013; Qing et al., 2018; Dennog et al., 1999). Nonetheless, comparative studies are scarce, and inter-species differences remain underexplored.

### 5.2 Across tissues and cell types

The proteins induced by HBO vary across different tissues. HO-1 and HSP70 are recognized as the most common and stable proteins induced by HBO, and their levels increase in the brain, spinal cord, myocardium, lungs, liver, and kidneys (Li et al., 2007; He et al., 2011; Huang et al., 2016; Bai et al., 2018; Xue et al., 2016; Liu et al., 2025; Huang et al., 2014; Yin et al., 2015; Yu et al., 2016; Hu et al., 2018; Soejima et al., 2012; Godman et al., 2010; Nesovic et al., 2021; Kovacevic et al., 2021; Chang et al., 2006; Liu et al., 2011). Besides HO-1 and HSP70, HBO also induces antioxidant proteins such as SOD, GCL, γGT, MRP1, xCT, GPX4, GSH (Li et al., 2007; Yan et al., 2013; Xue et al., 2016; Xu et al., 2014; Soejima et al., 2012; Nesovic et al., 2021; Kovacevic et al., 2021; Nie et al., 2006) and HSP90/27 (Huang et al., 2014; Ni et al., 2013; Shyu et al., 2004; Lin et al., 2012), as well as EPO, COX-2, MMP-2/9 proteins in the brain and spinal cord (Soejima et al., 2013; Cheng et al., 2011; Jadhav et al., 2009; Gu et al., 2008; Peng et al., 2008). HBO can also upregulate HSP27 and VEGF expression in myocardial tissue (Jeysen et al., 2011), as well as SOD, NQO1, TRX1 (Bai et al., 2018; Yu et al., 2016), and HSP27/40 (Xu et al., 2025; Ni et al., 2013; Tsai et al., 2014) in lung tissue. In liver tissue, HBO increases the expression of HSP70 and VEGF proteins (Yu et al., 2016), and in kidney tissue, HBO leads to the upregulation of SOD and CXCR4 proteins (Nesovic et al., 2024). The majority of these tissue-specific upregulated proteins are downstream targets of Nrf2, suggesting that this transcription factor plays a dominant role in protein expression in the tissues induced by HBO.

The induction of HO-1 and HSP70 is also commonly observed following HBO across diverse cell types. In the brain and spinal cord, HBO upregulates HO-1, SOD, and HSP70/90/27 in neurons (Li et al., 2007; Huang et al., 2016; Huang et al., 2014), and enhances HO-1, GCL, γGT, and MRP1 in astrocytes (Li et al., 2007; Xu et al., 2014; Hu et al., 2018). In pulmonary vascular endothelial cells, HBO stimulates expression of HO-1, NQO1, TRX1, HSP90/27/40, and VEGF (Xu et al., 2025; Papaiahgari et al., 2006; Godman et al., 2010). HBO can also elevate HSP70/90 and CXCR4 in peripheral blood mononuclear cells and peripheral lymphocytes (Fratantonio et al., 2021; Qing et al., 2018;). A high degree of overlap was observed between cellular and tissue induction profiles: neuronal and astrocytic expression patterns closely mirrored the tissues of central nervous system. In contrast, pulmonary endothelial cell expression recapitulated the pattern observed in lung tissue. These results confirm that HBO-induced proteins are consistent *in vitro* and *in vivo*, for HBO *in vitro* experiments.

### 5.3 Dynamic changes in protein expression

#### 5.3.1 *In vivo* expression

**FIGURE 2:**
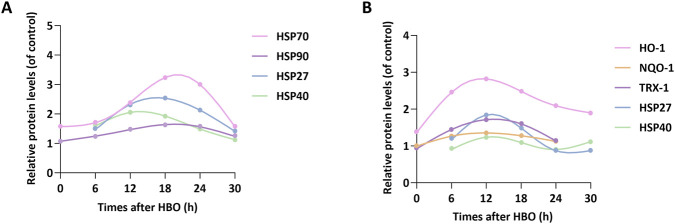
Temporal profiles of protein expression induced by HBO. Proteins were significantly upregulated *in vivo* and *in vitro*. Protein expression was quantified by Western blot and normalized to control values **(A)** illustrates temporal expression profiles in lung and spinal cord tissues, and **(B)** shows the consistent temporal induction trend observed across neurons, astrocytes, and pulmonary microvascular endothelial cells. The data are from published (Zhou et al., 2018; Huang et al., 2016; Xu et al., 2014; Liu et al., 2025; Huang et al., 2014; Ni et al., 2013; Qing et al., 2018; Zhou et al., 2021) and unpublished studies.

As shown in Figure 2A, we observed the expression of protective proteins at 6-h intervals over a 30-h period following HBO exposure (2.8 ATA, 60 min). Data from the spinal cord and lung indicated that the expressions of HSP70, HSP90, HSP27, and HSP40 reached their peaks within 12–20 h after HBO exposure. Among them, HSP40 peaked at 12 h (Xu et al., 2025), HSP27 peaked at 16–18 h (Xu et al., 2025), HSP70 peaked at 18–20 h (Ni et al., 2013; Qing et al., 2018), and HSP90 peaked at 18–24 h (Ni et al., 2013; Qing et al., 2018). In general, the highest expression of these proteins in the body occurred around 18 h after HBO. This provides a temporal reference for optimizing preconditioning timing in future human applications.

#### 5.3.2 *In vitro* expression

At the cellular level, the expression of HBO-induced proteins in neurons, astrocytes, and pulmonary microvascular endothelial cells peaked between 6 and 18 h (Figure 2B). Specifically, HO-1 and NQO1 expression peaked at 6–12 h, whereas TRX1 and HSP27/40 expression peaked later, at 12–18 h (Xu et al., 2025; Huang et al., 2014). These data indicate that the expression peaks of these protective proteins in cells occur around 12 h after HBO, defining an optimal time window for subsequent *in vitro* experimental design.

## 6 Protocol for HBO preconditioning

### 6.1 Pressure

HBO pressure is a critical determinant of the expression level of protective proteins. The expression of HSP70 was upregulated to 2.0–2.5-fold after HBO by 2.0 ATA for 60 min compared to the control (Tsai et al., 2014), whereas an approximately 4-fold increase was observed after a 2.8 ATA for 60 min HBO (Ni et al., 2013). Similarly, a 2-fold increase in HO-1 protein was observed with 2.0 ATA for 60 min of HBO exposure (), and a 3-fold rise was observed after 2.8 ATA for 60 min of HBO exposure (Huang et al., 2014). As HBO pressure increases, the expression of protective proteins also increases. However, this increase is not unlimited. When the pressure exceeds 4.8 ATA, HO-1 protein expression tends to stabilize and remains at 2.5–3.5-fold. This is attributable to the MEK1/2-mediated negative regulation of HO-1 (Huang et al., 2016). Furthermore, ROS production and transcription factor activity also rise with increasing HBO pressure (Zhou, 2018). The blood ROS level of athletes exposed to 2.5 ATA HBO was significantly higher than that of the 1.4 ATA group (Leveque et al., 2023). Other experiments also showed that the exposure pressure from 1.0 ATA to 1.4 ATA could enhance the transcriptional activity of HIF-1α and Nrf2 in human monocytes (Fratantonio et al., 2021). The above data indicate that within a certain pressure range, protective protein expression increases in direct proportion to the elevated HBO pressure. In consideration of the exposure pressures utilized for clinical HBO preconditioning, we propose the following protocol. A 5-min air break should be included between each oxygen inhalation session.

1. For 2.5–2.8 ATA, adopt 2 to 3 HBO sessions, each of 20 min.
2. For 2.2–2.5 ATA, adopt 4 HBO sessions, each of 20 min.
3. For 2.0–2.2 ATA, adopt 2 to 3 HBO sessions, each of 30 min.

### 6.2 Duration

While pressure optimizes protein expression, defining the optimal exposure duration is equally important. Data regarding the duration-dependent efficacy, however, were conflicting. Exposure to hyperoxia from 60 to 180 min has been shown to enhance Nrf2 DNA-binding activity and downstream target gene transcription of HO-1 and NQO1 (Papaiahga et al., 2004). At the same time, some evidence suggested that extending the exposure duration from 45 to 120 min could not further increase the level of hepatic SOD, GSH, or CAT (Yu et al., 2005). HBO sessions are typically limited to 40–90 min to minimize the risk of oxygen toxicity (Bhutani and Vishwanath, 2012), and preconditioning protocols therefore adhere to the same duration.

### 6.3 Repetition

Repeated HBO exposure is a common HBO treatment approach and also confers preconditioning benefits. Compared with three HBO sessions, five sessions can significantly enhance neuroprotective effects and improve ischemic tolerance in the rabbit spinal cord (Dong et al., 2002). Secondly, five sessions can increase HSP72 expression in hamster hippocampus and improve neuronal ischemic tolerance compared with three sessions (Wad et al., 1996; Dong et al., 2002; Xiong et al., 2000; Ostrowski et al., 2008). Another study suggested that a single HBO session is sufficient to protect rats’ livers from subsequent I/R injury (Yu et al., 2005). Our research also found that a single HBO pretreatment can reduce the incidence of DCS in rats and increase expression of HSP70, HO-1, and HSP70/40/27 (Xu et al., 2025; Huang et al., 2014; Ni et al., 2013; Qing et al., 2018). It is believed that whether increasing HBO exposure frequency can provide additional protective benefits depends on specific circumstances. When dealing with high incidence rates or potential injuries with sufficient preparation time, such as organ transplantation or other anticipated operations, repeated HBO stimulation can enhance the protective effect. However, for potential injuries with low incidence rates or insufficient preparation time, such as preventing diving-related diseases, a single HBO session can already provide sufficient protective effects. It should be noted that various factors, including the injured tissue and the type of injury, also influence the protective effect of HBO. Therefore, more clinical evidence is needed to optimize the HBO preconditioning protocol.

## 7 Application of HBO preconditioning in disease

**FIGURE 3:**
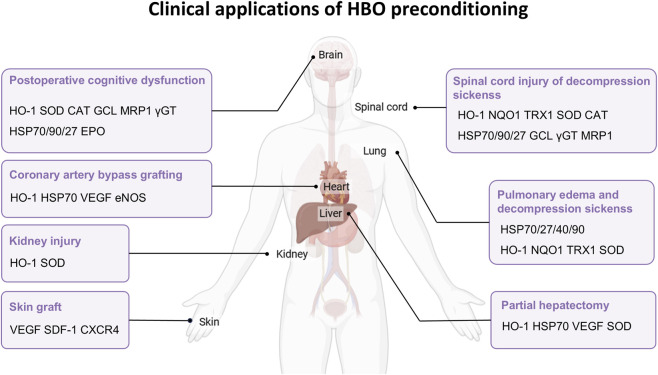
Clinical applications of HBO preconditioning. Current clinical applications of HBO across human diseases, including cardiovascular, neurological, pulmonary, and hepatic diseases, and prospects for its use in kidney injury and skin grafts. The figure lists the key proteins in each disease.

Extensive research has established HBO preconditioning as a preventive strategy for various diseases. In some cases, preclinical findings have been successfully translated into clinical applications (Figure 3).

### 7.1 Cardiovascular diseases

#### 7.1.1 Clinical applications

HBO as a prophylactic approach is first supported by a prospective randomized double-blind trial of coronary artery bypass grafting (CABG) (Alex et al., 2005). In this study, 64 CABG patients were randomized to receive three sessions of HBO (2.4 ATA, 60min) at 4, 12, and 24 h before surgery. HBO preconditioning significantly upregulated postoperative HSP70 expression, reduced CD18 and TNF-α, and decreased the systemic inflammatory response after surgery (Alex et al., 2005). Yang Li et al. reported that the cardioprotective effect has also been verified in CABG patients who underwent 5 HBO sessions (2.0 ATA, 70min), as evidenced by a lower serum troponin I level during the perioperative period, and a cardiac protective effect was observed (Li et al., 2011a). In another study, a single HBO (2.4 ATA, 60min) 2 h before the surgery could induce the expression of myocardial eNOS and HSP70 proteins during I/R and improve postoperative left ventricular stroke work (Jeysen et al., 2011). HBO pretreatment could also reduce the occurrence of surgical complications and postoperative complications, reduce the use of positive inotropic drugs, and effectively shorten the postoperative ICU stay (Yogaratnam et al., 2010).

#### 7.1.2 Fundamental research

Evidence from animal studies explores the efficacy of HBO preconditioning in mitigating myocardial I/R injury (Kim et al., 2001; Yogaratnam et al., 2008). It has been shown that PI3K/Akt/Nrf2 pathway was induced by HBO to combat myocardial I/R injury in a mouse model (). Furthermore, the anti-inflammatory and anti-autophagic effects of HBO have been identified as exerting cardioprotection in rats with myocardial I/R injury (Chen et al., 2017).

### 7.2 Neurological diseases

#### 7.2.1 Clinical applications

The clinical benefits of HBO pretreatment for the nervous system mainly center on reducing postoperative cognitive dysfunction (POCD). A study has shown that a single HBO session (2.4 ATA, 60 min) can reduce the incidence of neurological complications after CABG surgery (Yogaratnam et al., 2010). Furthermore, three HBO sessions (2.4 ATA - 60 min) before CABG surgery significantly reduce the incidence of postoperative POCD (Alex et al., 2005). Five HBO treatments before CABG surgery similarly yield significant reductions in perioperative nerve injury markers S100B protein and NSE, as well as in the incidence of POCD in patients after surgery (Li et al., 2011a; Li et al., 2011b; Zhao et al., 2019). Beyond the above studies, many similar studies are reporting consistent findings (Li, 2022; Wu et al., 2015). Besides, HBO pretreatment can also reduce the incidence of POCD in elderly hip replacement surgery patients (Fu et al., 2019).

#### 7.2.2 Fundamental research

HBO can enhance the ischemic tolerance of animal models after cerebral I/R injury (Soejima et al., 2013; Hentia et al., 2018; Wu et al., 2023; Wang et al., 2021; Miljkovic-Lolic et al., 2003). It increases the levels of beneficial proteins HO-1, GST, and EPO, and reduces the levels of pro-inflammatory proteins COX-2 and HMGB1 in the brain (Wu et al., 2023). Studies have shown that HBO-induced Sirt1 activation of Nrf2-dependent antioxidant defenses and suppression of apoptosis by deacetylating p53 in rats with focal cerebral ischemia (Xue et al., 2016; Alcendor et al., 2004). HBO-induced osteopontin also contributes to reducing POCD in rats with ischemic stroke (Hu et al., 2015). HBO exerts neuroprotective effects in animal and cell models of spinal cord I/R by upregulating protective proteins such as HO-1, Mn-SOD, CAT, and Bcl-2, thereby enhancing spinal cord ischemia tolerance (Huang et al., 2016; Huang et al., 2014; Nie et al., 2006; Dong et al., 2002; Wang et al., 2009).

### 7.3 Respiratory diseases

#### 7.3.1 Clinical applications

For individuals preparing to enter high-altitude regions, HBO enhances antioxidant activity by elevating SOD and reducing MDA, thereby reducing the incidence of acute altitude reaction (Chen and Zhou, 2008). HBO preconditioning alleviates the body’s hypoxic response at high altitudes, improves cognitive and physical function, and prevents acute mountain sickness (AMS) (You et al., 2023). You et al. summarized dozens of clinical studies on HBO for the prevention of AMS. The mechanisms include increasing blood oxygen saturation and arterial oxygen pressure, and increasing plasma dopamine, adrenaline, and adrenocorticotropic hormone to accelerate the establishment of a new internal balance (You et al., 2023). Furthermore, HBO pretreatment improves cognitive functions such as memory and responsiveness, and increases energy metabolism in individuals under physical load at high altitude, thus alleviating fatigue and enhancing work efficiency (You et al., 2023).

#### 7.3.2 Fundamental research

The relevant animal studies mainly focused on preventing high-altitude pulmonary edema (You et al., 2023). HBO can upregulate the expression of HSP70 and AQP1/5, thereby alleviating pulmonary edema, bleeding, and inflammation in rats (Tsai et al., 2014). Moreover, HBO has also been used to protect against acute lung injury in rat models by promoting Nrf2 nuclear import and inducing HO-1 and NQO1 (Bai et al., 2018; Yu et al., 2016).

### 7.4 Gastrointestinal disorders

#### 7.4.1 Clinical applications

In a prospective, randomized, double-blind trial, 10 patients undergoing pancreaticoduodenectomy who received a single HBO session (2.5 ATA, 82 min) 24 h before resection had lower systemic inflammation and pulmonary infection rates than those without HBO (Bosco et al., 2014).

#### 7.4.2 Fundamental research

In animal models of hepatic I/R injury, HBO was observed to upregulate HSP70 and HO-1 expression in liver tissue, thereby alleviating inflammation and oxidative damage and preserving liver function. It has been reported that HIF‐1α and VEGF in rat liver are increased by HBO pretreatment, thereby enhancing hepatic revascularization and improving postoperative survival after partial hepatectomy (). Moreover, in rat models of intestinal I/R injury and colonic anastomosis, HBO enhanced SOD and GSH-Px levels, prevented apoptosis, and accelerated wound healing (Poyrazoglu et al., 2015; Bertoletto et al., 2008).

### 7.5 Urological diseases

#### 7.5.1 Clinical applications

To date, research on HBO for the prevention of urological diseases in humans remains absent.

#### 7.5.2 Fundamental research

HBO has a protective effect on renal I/R injury. Animal experiments have shown that upregulation of HO-1, SOD, and Bcl-2 induced by HBO reinforces antioxidant capacity, reduces lipid peroxidation, and improves renal haemodynamics and functional recovery (He et al., 2011; Nesovic et al., 2021; Kovacevic et al., 2021; Ivanov et al., 2020; Kovacevic et al., 2020). HBO also elevated renal HIF-1α and suppressed NF-κB activation in rat models of postischemic acute kidney injury, thereby limiting inflammation and promoting repair (Nesovic et al., 2024).

### 7.6 Decompression sickness

#### 7.6.1 Clinical applications

The role of preoxygenation in DCS prevention has been extensively studied before diving, submarine rescue operations, high-altitude decompression, and extravehicular activities in space (Gempp and Blatteau, 2010). Landolfi et al. found that prophylactic HBO reduced bubble formation and platelet activation in divers (Landolfi et al., 2006). Preoxygenation 30 min before diving reduced bubble formation during repeated dives, and this protective effect was observed after the first dive and persisted into the second dive, even when no preoxygenation was performed before the second dive. These results suggest that the protective effect of preoxygenation is not merely due to denitrogenation and denucleation, but also to the activation of protective proteins in the body (Castagna et al., 2009). To date, however, no study has reported the effects of HBO on protective proteins in divers.

#### 7.6.2 Fundamental research

Several studies have shown that HBO preconditioning reduced the occurrence of DCS in animals (Butler et al., 2006; Gennser and Blogg, 2008). We performed a single HBO session at 2.8 ATA for 60 min before simulated diving and found a reduction in both the incidence and mortality of DCS in rats (Fan et al., 2010). Further mechanistic studies revealed that this beneficial effect was attributed to moderate ROS induction, which activated Nrf2 and HSF-1, leading to upregulation of HO-1, TRX1, NQO1, and HSP70/40/27 (Huang et al., 2016; Xu et al., 2025; Xu et al., 2014; Huang et al., 2014; Ni et al., 2013). Based on the existing evidence and proposed mechanism, HBO could be integrated into pre-dive compression exercises or surface decompression procedures for challenging diving activities.

### 7.7 Skin graft

#### 7.7.1 Clinical applications

To date, clinical trials on HBO preconditioning for skin graft are absent.

#### 7.7.2 Fundamental research

A number of animal studies have evaluated the effect of HBO preconditioning on ischemia-reperfusion injury of skin flaps, assessed by flap survival rate, flap size, and average blood perfusion. It has been reported that HBO promoted neovascularization by increasing the expression levels of HIF-1α, VEGF, SDF-1, and CXCR4 in transplanted rat skin flaps (Liu et al., 2014). Additionally, HBO pretreatment mitigated rat flap I/R injury by regulating apoptosis through raising Bcl-2 while reducing Bax and Caspase-3 (Xiao et al., 2015), and inflammatory responses by inhibiting the expression of ICAM-1 and VCAM-1 (Song et al., 2016; Qi et al., 2013). These studies indicate that HBO could potentially prevent I/R injury of transplanted skin flaps.

## 8 Conclusion

HBO preconditioning, as a promising preventive measure, primarily functions by activating transcription factors Nrf2, HSF1, and HIF-1α, and their downstream proteins such as HO-1, HSP70, and VEGF, through the production of moderate ROS, thereby enhancing tissue tolerance to various stresses, including ischemia and hypoxia. Based on the HBO treatment protocol and existing research, we recommend a 2.5 ATA, 60 min (interrupted by two 5 min air intervals), or 80 min (interrupted by three 5 min air intervals) exposure protocol as a safe and effective pretreatment plan. We also clarified the temporal characteristics of protein expression, which peaks *in vivo* approximately 18 h after HBO exposure and *in vitro* at 12 h. This temporal profile provides a key basis for determining the optimal time window for pretreatment intervention in clinical practice. It offers an important reference for the exploration of protein regulation under physiological and pathological conditions in cellular research. To date, HBO preconditioning has been implemented in the preoperative management of CABG patients, and emerging evidence suggests its potential utility in other high-risk surgeries. In the future, more basic research and clinical observations are still needed to translate this prophylactic measure into clinical practice.

## Glossary

AKT: Serine/threonine kinase
AQP: Aquaporin
ARE: Antioxidant response element
Bcl-2: B Cell lymphoma-2
Bax: Bcl-2-associated X protein
CABG: Coronary artery bypass grafting
CAT: Catalase;
Cul3: Cullin-3
CXCR4: C-X-C motif chemokine receptor four
DCS: Decompression sickness
EPO: Erythropoietin
ERK: Extracellular signal‐regulated kinases
FIH: Factor inhibiting HIF
GCL: Glutamate-cysteine ligase
GPX4: Glutathione peroxidase four
GSH: Glutathione
HBO: Hyperbaric oxygen
HIF-1: Hypoxia-inducible factor 1
HMGB1: High Mobility Group Box 1 protein
HO-1: Heme oxygenase-1
HRE: Hypoxia response element
HSE: Heat shock element
HSP: Heat shock protein
HSF1: Heat shock factor 1
ICAM-1: Intercellular Adhesion Molecule-1
I/R: Ischemia-reperfusion
JNK: c-Jun N-terminal kinase
Keap1: Kelch-like ECH-associated protein 1
MAPK: Mitogen-activated protein kinase
MEK1/2: Mitogen-activated extracellular signal-regulated kinase 1 and 2
MMPs: serine/threonine kinase
MRP1: Multidrug resistance-associated protein 1
NBO: Normobaric hyperoxia
NF-κB: Nuclear Factor kappa-light-chain-enhancer of activated B cells
NOS: Nitric Oxide Synthase
NQO1: NAD(P)H: quinone oxidoreductase-1
Nrf2: Nuclear factor erythroid 2-related factor 2
POCD: Postoperative cognitive dysfunction
p38 MAPK: p38 mitogen-activated protein kinase
PHD: Prolyl hydroxylase domain
PI3K: Phosphoinositide 3-kinase
ROS: Reactive Oxygen Species
Sirt1: Sirtuin 1
SLC7A11: Solute carrier family seven member 11
SOD: Superoxide dismutase
STAT3: Signal transducer and activator of transcription 3
TNF-α: Tumor Necrosis Factor-α
TRX1: Thioredoxin-1
VCAM-1: Vascular Cell Adhesion Molecule-1
VEGF: Vascular endothelial growth factor
γ-GT: Gamma-glutamyl transferase
